# Human Kidney‐Derived Cells Ameliorate Acute Kidney Injury Without Engrafting into Renal Tissue

**DOI:** 10.1002/sctm.16-0352

**Published:** 2017-04-04

**Authors:** Ilaria Santeramo, Zeneida Herrera Perez, Ana Illera, Arthur Taylor, Simon Kenny, Patricia Murray, Bettina Wilm, Norbert Gretz

**Affiliations:** ^1^ Department of Cellular and Molecular PhysiologyCentre for Preclinical Imaging, Institute of Translational Medicine, the University of LiverpoolLiverpoolUnited Kingdom; ^2^ Medical Research Center, Medical Faculty Mannheim, University of HeidelbergHeidelbergGermany; ^3^ Department of Paediatric Surgery and UrologyAlder Hey Children's NHS TrustLiverpoolUnited Kingdom

**Keywords:** Cisplatin‐induced nephropathy in nude rats, Human kidney progenitor cells, CD133, Regenerative medicine therapies, Transcutaneous glomerular filtration rate measurement

## Abstract

Previous studies have suggested that CD133^+^ cells isolated from human kidney biopsies have the potential to ameliorate injury following intravenous (IV) administration in rodent models of kidney disease by integrating into damaged renal tissue and generating specialized renal cells. However, whether renal engraftment of CD133^+^ cells is a prerequisite for ameliorating injury has not yet been unequivocally resolved. Here, we have established a cisplatin‐induced nephropathy model in immunodeficient rats to assess the efficacy of CD133^+^ human kidney cells in restoring renal health, and to determine the fate of these cells after systemic administration. Specifically, following IV administration, we evaluated the impact of the CD133^+^ cells on renal function by undertaking longitudinal measurements of the glomerular filtration rate using a novel transcutaneous device. Using histological assays, we assessed whether the human kidney cells could promote renal regeneration, and if this was related to their ability to integrate into the damaged kidneys. Our results show that both CD133^+^ and CD133^−^ cells improve renal function and promote renal regeneration to a similar degree. However, this was not associated with engraftment of the cells into the kidneys. Instead, after IV administration, both cell types were exclusively located in the lungs, and had disappeared by 24 hours. Our data therefore indicate that renal repair is not mediated by CD133^+^ cells homing to the kidneys and generating specialized renal cells. Instead, renal repair is likely to be mediated by paracrine or endocrine factors. Stem Cells Translational Medicine *2017;6:1373–1384*

**Significance Statement**

This study shows that human kidney cells ameliorate renal injury following intravenous injection in rats. Despite significantly improving renal function and ameliorating tissue damage, the cells do not engraft in the kidney, instead being entrapped in the lungs where they rapidly undergo cell death. These findings indicate that the therapeutic effects of the cells are mediated by secreted factors, possibly released by the dying cells. This raises the possibility that in the future, therapies for kidney disease could be based on cell‐derived factors rather than on the cells themselves, thereby circumventing the risks associated with cell administration, such as tumor formation.

## Introduction

Acute kidney injury (AKI) is associated with an abrupt decline in renal function, and is reported to have a mortality rate ranging from 18% to 80% 1, 2. The severity and duration of the acute injury correlates with the incidence of progression to chronic or even end stage renal disease (ESRD) 3. Successful therapeutic interventions of AKI may not only promote recovery from acute injury, but could also decrease the incidence of ESRD.

Cisplatin is a chemotherapy drug that is used to treat a wide range of solid tumors 4, but can cause severe nephrotoxicity, which is associated with 20%–40% reduction in the glomerular filtration rate (GFR), increased serum urea, elevated serum creatinine (sCr) and acute tubular necrosis 5, 6.

In rodent models, cisplatin‐induced AKI is routinely used to test the efficacy of novel therapies 7, 8, 9, 10, 11, including a range of cell‐based regenerative medicine therapies 12. Studies which tested the efficacy of mesenchymal stem/stromal cells (MSCs) showed that these cells promote repair through the secretion of paracrine factors that stimulate the regeneration of host renal tissue 12, 13, 14. In contrast, nephron progenitor cells derived from human fetal kidneys appear to improve renal health by integrating into damaged rodent kidneys and generating specialized renal cells 15.

Recently, a population of putative progenitor cells expressing the glycosylated isoform of CD133 (Prominin‐1) was identified in human adult kidneys 16, 17. CD133 is a marker of cancer stem cells and has been used to isolate stem and progenitor cells from several tissues 18. Human adult kidney‐derived CD133^+^ cells have been shown to ameliorate injury following intravenous (IV) administration in mouse models of glycerol‐induced rhabdomyolysis 16, 19, 20 and adriamycin‐induced nephropathy 17. Specifically, it was suggested that CD133^+^ cells ameliorate injury by engrafting into the kidneys and differentiating into specialized renal cells 17, 19. These observations have raised the prospect of developing autologous cell therapies to replace damaged renal tissue in patients with kidney disease.

In order to track the fate of exogenous cells in preclinical models, cells are frequently labeled using membrane‐bound dyes in order to allow their detection in histological sections at the study end point 17, 19, 21. In previous studies assessing the role of CD133^+^ kidney‐derived cells in ameliorating renal injury, cells labeled with the lipophilic fluorescent dye, PKH26, appeared to engraft in the kidney following IV administration and express markers of podocytes and proximal tubule cells (PTCs) 17, 19, 21. However, PKH26 has been shown to be an unreliable tracking agent because it can be transferred to host cells, leading to false positive results 22, 23.

In this study, we aimed to accurately assess the improvement of renal function in response to CD133^+^ human kidney cell administration in rats after cisplatin‐induced AKI, and compare the therapeutic efficacy of the CD133^+^ cells with a negative control population of CD133^−^ cells. The GFR is the most accurate measure of renal excretory function but classical GFR measurements involve the need for repeated blood and/or continuous urine sampling over a prolonged time period (5–24 hours), which is difficult to obtain in rodent models. In order to monitor kidney function, we used a novel transcutaneous device to determine longitudinally the half‐life (*t*_1/2_) of fluorescein isothiocyanate (FITC)‐sinistrin, a molecule that is exclusively filtered by the kidneys, as a measure of the GFR 24, 25, 26, 27. The device has recently been used by our group to monitor renal function in a mouse adriamycin model, where it was found to be a good predictor of histological damage 28. In the current study, we measured FITC‐sinistrin *t*_1/2_ before cisplatin administration, before cell injection and at several points up to day 14 in rats administered with either CD133^+^ or CD133^−^ human kidney cells, or saline (control group), to determine for each individual animal the extent by which the cell treatment affected kidney function.

A further aim was to identify any association between therapeutic efficacy and the extent of renal engraftment. To address this question, we introduced a green fluorescent protein (GFP) reporter gene into the CD133^+^ and CD133^−^ cells so that their location within specific organs could be analyzed histologically. We also labeled the cells with the lipophilic dye, PKH26, to investigate whether there was any propensity for this dye to separate from the GFP^+^ kidney cells and label host cells, thereby giving false positive results.

## Materials and Methods

### Isolation and Lentiviral Transduction of Human Kidney Cells

Infant renal tissue was obtained from kidneys deemed unsuitable for transplantation via UK National Health Service Blood and Transfusion (NHSBT, www.nhsbt.nhs.uk/). The tissue was minced in small pieces in sterile conditions and digested with collagenase I (1 mg/ml, C0130, Sigma, www.sigmaaldrich.com) for 90 minutes at 37°C, centrifuged and incubated with Dulbecco's Modified Eagle's Medium/F12 containing DNAse I (1%) (Sigma) for 15 minutes at room temperature. The cell suspension was filtered through a 70‐µm and a 40‐µm sterile sieve and plated in the human renal progenitor cell medium previously used for fetal progenitor cells 29. The medium was changed every 2 days until the cells reached 90% confluence.

Lentiviral particles were produced as previously described 30 using an unmodified eGFP vector (pHIV‐eGFP, 21373 Addgene, www.addgene.org) with a multiplicity of infection (MOI) of 5, yielding >90% labeling efficiency. Typically, 2 × 10^5^ cells were plated 8 hours before transduction. Once attached, the cells were incubated for 16 hours in complete medium containing the appropriate amount of lentiviral particles containing 8 µg/ml of Polybrene (H9268 Sigma). The medium was then appropriately discarded, and the cells were grown in normal medium.

Details regarding cell sorting, cell characterization and immunofluorescence protocols are provided in the Supporting Information methods section.

### Animals

All in vivo experiments were conducted at the University of Heidelberg in accordance with the German Animal Protection Law and approved by the local authority (Regierungspräsidium Nordbaden, Karlsruhe, Germany, in agreement with EU directive 2010/63/EU). Eight‐ to nine‐weeks‐old male immunodeficient athymic nude rats (Crl:NIH‐Foxn1^rnu^, Charles River Laboratories, www.criver.com/) were housed in pairs in individually ventilated cages and acclimated for 1 week before the start of the experiments.

### Cisplatin‐Induced Kidney Injury

Freshly prepared cisplatin solution (Sigma), dissolved in sterile 0.9% saline (AlleMan Pharma, deltamedica.de/) was administered intraperitoneally (IP) at 7 mg/100 g body weight. For the 2‐week experiment, a total of 20 male immunodeficient athymic nude rats were used. Onset of renal damage following cisplatin administration was confirmed at day 2 via measurements of FITC‐sinistrin (Fresenius Kabi, Linz, Austria, www.fresenius-kabi.com) *t*_1/2_ as described below.

### Renal Biomarker Analysis

Serum was collected from whole blood samples before the induction of damage, and then on days 7 and 14 after cisplatin administration via the ophthalmic venous plexus (orbital sinus), and stored at −20°C until use. For urine collection, the animals were housed for 16 hours in individual metabolic cages with free access to water and food. At the end of each collection period, the urine volume was recorded, and the samples were centrifuged (78 g, 5 minutes) and immediately frozen at −20°C until analyzed. sCr, serum urea and urine creatinine were determined using the Cobas c311 analyzer (Roche Diagnostics GmbH, Mannheim, Germany, www.roche.com). Albumin levels in urine were determined by ELISA and the values were normalized to the amount of urinary creatinine over a 24‐hour period. Assessment of glomerular filtration at baseline and on days 2, 7, and 14 after cisplatin administration was performed by administering FITC‐sinistrin into the tail vein, and detecting the fluorescence signal transcutaneously for 90 minutes using a miniaturized device (Mannheim Pharma and Diagnostic, Mannheim, Germany, www.medibeacon.com/). The FITC‐sinistrin *t*_1/2_ was computed using a specifically designed software 31.

### Cell Administration

Before each injection, GFP‐expressing CD133^+^ and CD133^−^ cell populations were incubated with PKH26 (Sigma), following manufacturer's instructions. Animals that displayed renal injury by day 2 following cisplatin administration (as indicated by the FITC‐sinistrin *t*_1/2_ measurements) were randomly assigned to three groups, two of which received either 10^6^ GFP‐expressing CD133^+^ or CD133^−^ cells in a 500 µl volume of sterile phosphate‐buffered saline (PBS) via lateral tail vein injection; the cisplatin‐injured control group received PBS (CD133^+^ group, 6 animals; CD133^−^ group, 6 animals; cisplatin‐injured control group, 7 animals). A scheme of the experimental design is shown in Fig. 2A). On day 7, cell‐treated animals received a second dose of either 10^6^ GFP‐expressing CD133^+^ or CD133^−^ cells into the tail vein.

For the biodistribution study, a total of eight male immunodeficient athymic nude rats were used. After assessment of cisplatin‐induced renal damage on day 2, 10^6^ CD133^+^ cells were injected into the lateral tail vein of 6 animals. 2 animals each were sacrificed at 1, 6, and 24 hours after cell injection. Additionally, 2 uninjured control animals were sacrificed 1 hour after injection of saline.

### Morphological and Histological Analysis

For the morphological and histological analysis, four uninjured rats were included in the study. Immediately after culling, kidneys and lungs were excised, fixed in 4% paraformaldehyde for 24 hours and paraffin embedded or frozen for immunohistological analysis (see below). Three micrometer‐thick paraffin sections were stained with hematoxylin and eosin (H&E) or Masson's Trichrome (MT). For the measurement of mean luminal area, 10 random fields of the renal cortex of each animal were imaged using a ×20 objective. The images were transformed into black and white (B&W, 8 bit), and a B&W threshold was applied using Fiji software. The luminal areas on the edge of the image were excluded from the analysis. For measurement of the fibrotic areas in MT‐stained sections, 10 random fields of the renal cortex of each animal were imaged using a ×20 objective. A color threshold (blue) to all images was applied using Fiji software and the blue area was computed. Also, 5 to 7 MT images of two animals per group were stitched together using the “grid‐collection stitching” plug‐in of the Fiji software.

### Immunostaining of Tissue Sections

Frozen sections were blocked with 0.1% Triton X‐100 (Sigma) and 10% goat serum (Sigma) in PBS and incubated with anti‐Calbindin (1:500, Sigma, C9848), anti‐CD68 (1:500, Abcam, www.abcam.com, ab31630) anti‐GFP (1:5,000, Abcam, ab6556), anti‐HLA (1:50, Santa Cruz Biotechnology, www.scbt.com, sc‐25619), anti‐human nuclei (1:200, Millipore, www.merckmillipore.com, MAB 1281), anti‐IL10 (1:200, Abcam, ab9969), anti‐megalin (1:200, Acris antibodies, www.acris-antibodies.com, DM3613P) followed by incubation with the secondary antibodies Alexa Fluor488‐coupled goat anti‐rabbit IgG, Alexa Fluor 633‐coupled goat anti‐mouse IgG_1,_ Alexa Fluor 647‐coupled goat anti‐rabbit, Alexa Fluor 594‐coupled goat anti‐mouse IgG_1_ (Thermo Fisher Scientific, www.thermofisher.com). Nuclei were counterstained with 4′,6‐Diamidino‐2‐Phenylindole, Dihydrochloride (Thermo Fisher Scientific). Fluorescence images were taken using a spinning disk confocal microscope CSU‐X1 (3i Marianas, www.intelligent-imaging.com), coupled with a digital camera (CMOS, Hamamatsu, www.hamamatsu.com). The megalin stitched images were composed using the “pairwise stitching” plug‐in of the Fiji software 32.

### Statistics

All data are presented as mean ± standard error mean (SEM). All statistical analysis was performed using GraphPad software, and a one‐way analysis of variance was used to compare three or more groups. The statistical significance was assumed for a *p* value (*p*) < .05.

## Results

### Human Kidney‐Derived Cells Express CD133 in Culture

**Figure 1:**
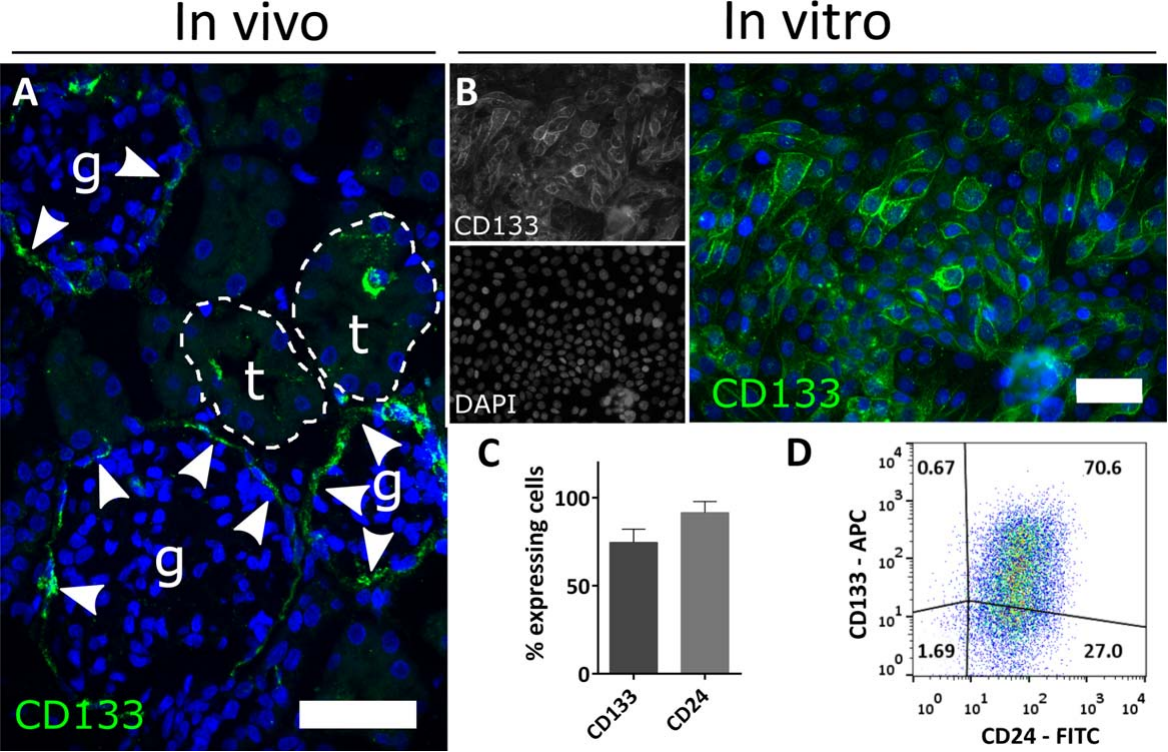
Identification and isolation of a population of human kidney cells. **(A)**: Representative confocal fluorescence images of human kidney cells from infant human renal tissue showing the expression pattern of CD133 within the Bowman's capsule (highlighted by white arrows) and on the apical surface of scattered tubular cells. **(B)**: Representative fluorescence images of bulk cultured cells at passage 1 after isolation, stained for CD133. Most of the cells appear CD133‐positive. **(C)**: FACS analysis showing the proportion of CD133^+^ and CD24^+^ cells within the bulk population at passage 2. The majority of the cells in the bulk population express CD133 (68.8% ± 9.2%) and CD24 (86.10% ± 6.3%). **(D)**: Representative flow cytometry Dot Plot of the bulk population at passage 2 stained with CD133 (APC) and CD24 (FITC) antibodies. Magnification: (A, B) ×400, scale bar 50 µm. Abbreviations: APC, allophycocyanin; DAPI, 4′,6‐diamidino‐2‐phenylindole; FITC, fluorescein isothiocyanate.

In order to analyze the role of human kidney‐derived cells in renal regeneration or repair, we generated primary cultures of renal cells by dissociating cortical fragments isolated from healthy infant renal tissue. We analyzed histological sections and primary renal cell cultures for expression of CD133, which has been previously described as a marker for kidney progenitor cells 16, 21, 33. Immunohistological analysis of the kidney sections demonstrated CD133 localization in cells of the Bowman's capsule, and on the apical surface of scattered tubular cells (Fig. 1A), similar to the pattern observed in adult human kidneys 17, 19, 34. Following tissue dissociation, more than 65% of the cells in the primary cultures expressed CD133, as shown by immunofluorescence (Fig. 1B) and flow cytometric analysis (Fig. 1C). Since CD133^+^ renal progenitor cells have been reported to coexpress CD24 35, we verified by flow cytometry that all CD133^+^ expressed CD24; however, only 70% of CD24^+^ cells expressed CD133 (Fig. 1D). Thus, our results show that following isolation, the majority of the kidney‐derived cells expressed CD133 in culture.

### CD133^+^ and CD133^−^ Human Kidney Cells Ameliorate Renal Function

**Figure 2:**
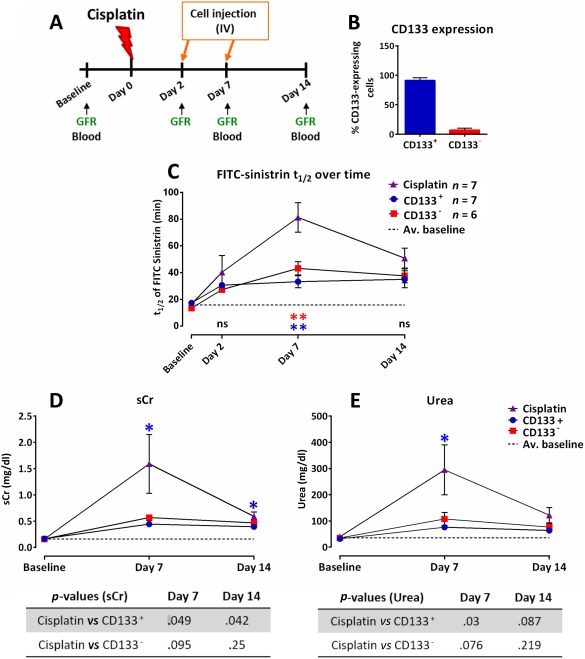
Both CD133^+^ and CD133^−^ human kidney cells can ameliorate renal function at day 7. **(A)**: Experimental design. **(B)**: FACS analysis of the expression of CD133 in both CD133^+^ and CD133^−^ populations at passage 5; mean values ± SEM of three independent sortings and expansions. **(C)**: Mean FITC‐sinistrin *t*_1/2_ values ± SEM, *p* = .0026 (blue), *p* = .0095 (red). **(D)**: Serum creatinine levels. **(E)**: Urea levels. The dotted line represents the average of the respective baseline values of all animals (*n* = 20). A one‐way ANOVA statistical test with Dunnet post hoc analysis was applied to each data set for each time point to compare the groups. *p* values are indicated in the tables below the graphs. CD133^+^ group (*n* = 6); CD133^−^ group (*n* = 6); cisplatin‐injured group (*n* = 7). Abbreviations: FITC, fluorescein isothiocyanate; GFR, glomerular filtration rate; ns, not significant; sCr, serum creatinine.

We induced kidney injury in 8‐ to 9‐week‐old male athymic nude rats by injecting cisplatin at 7 mg /100 g body weight. Animals were monitored for renal function by measuring the FITC‐sinistrin *t*_1/2_ at days 2, 7, and 14, and the serum injury markers sCr and urea at days 7 and 14. In 62.5% (20 out of 32) of the rats, an increase in the FITC‐sinistrin *t*_1/2_ was detected at day 2 when compared to baseline measurements before cisplatin administration. Only these animals were used for the subsequent study by assigning them to three groups which received on days 2 and 7 by IV injection either (a) CD133^+^ passage 5 (P5) cells, (b) CD133^−^ P5 cells, or (c) saline (Fig. 2A).

Prior to injection, the cells had been transduced with a pHIV‐eGFP vector and sorted for CD133 expression using fluorescence activated cell sorting (Supporting Information Fig. S1A–1D). Flow cytometry demonstrated that at passage 5, the GFP^+^ CD133^+^ population had a purity of 91.36% ± 9.17%, and the GFP^+^ CD133^−^ population had a purity of 92.99% ± 6.00% (*n* = 3) (Fig. 2B). The cell morphologies of the two populations after GFP‐lentivirus transduction were very similar to the un‐transduced CD133^+^ and CD133^−^ cells (Supporting Information Fig. S1E). The CD133^+^ population appeared epithelial‐like, whereas the CD133^−^ population was composed of elongated mesenchymal‐like cells. Compared with CD133^+^ cells, the CD133^−^ cells expressed noticeably lower levels of the epithelial marker, Epcam (Supporting Information Fig. S2). To investigate if the CD133^−^ cells resembled MSCs, which are known to reside in the kidney 36, we performed flow cytometric analysis to determine the expression levels of key MSC markers. We found that in comparison with human bone marrow‐derived MSCs, both the CC133^+^ and CD133^−^ cells expressed very low levels of the MSC markers, CD90 and CD105 (Supporting Information Fig. S2). Thus, given that the CD133^−^ cells are nonepithelial, and are also not MSCs, it is most likely that they are renal interstitial cells, which include various cell types, including interstitial fibroblasts 37. Prior to administering the cells, as an additional method to track them, the cells were labeled with the lipophilic fluorescent dye, PKH26, which had been used in previous studies 17, 19, 21, 38, 39.

Sequential measurements of the FITC‐sinistrin *t*_1/2_ clearance revealed that on day 7, the FITC‐sinistrin *t*_1/2_ was significantly reduced in animals that received either CD133^+^ or CD133^−^ cells, when compared to those of the cisplatin‐injured group, indicating an improvement in renal function (Fig. 2C; Supporting Information Table S1). It is important to note that no significant differences in FITC‐sinistrin *t*_1/2_ were observed between animals of the two cell‐treated groups. By day 14, the FITC‐sinistrin *t*_1/2_ had decreased in the cisplatin‐injured animals, and was no longer significantly different from that of the cell‐treated groups. No further decrease in the FITC‐sinistrin *t*_1/2_ was observed in the cell‐treated groups after day 7, indicating that the second cell injection had no additional beneficial effects on renal function (Fig. 2C).

In addition to the improvement in glomerular filtration function, the administration of CD133^+^ cells resulted in statistically reduced sCr (by 72.3%) and urea (by 74.2%) at day 7. In the CD133^−^‐treated group, sCr and urea values were also lower than in rats from the cisplatin‐injured group, though these were not statistically significant (Fig. 2D, 2E; Supporting Information Table S1). By comparing the FITC‐sinistrin *t*_1/2_ values at day 14 with the baseline values, it could be seen that renal function was not completely restored in either the CD133^+^ or CD133^−^ treated rats (Fig. 2C). The comparison between sCr baseline and d14 values also suggested that cell treatments failed to restore levels back to baseline (Fig. 2D), while the serum urea values suggested there was a complete restoration (Fig. 2E). However, given that both these biomarkers are less accurate indicators of renal function than FITC‐sinistrin *t*_1/2_, we conclude that the cell therapy does not fully restore renal function.

To further investigate the degree of tubular injury at the study end‐point (day 14), kidney sections were stained for the PTC marker, megalin, and the collecting tubule marker, calbindin. No differences in the pattern of calbindin staining was observed between any of the groups, but megalin staining was atypical in the cisplatin‐injured rats, consistent with PTC injury (Supporting Information Fig. S3A, S3B). It is known that megalin plays an important role in reabsorbing filtered albumin 40. Thus, to investigate if the injured rats displayed higher levels of albuminuria compared with those that received cell therapy, the urinary albumin:creatine ratio was measured at days 2, 7 and 14. The results showed that the albumin:creatinine ratio increased from day 7 to day 14 in the injured rats, but was not significantly greater than background levels in rats that received the cell therapy (Supporting Information Fig. S3C).

Thus, our results demonstrate that CD133^+^ cells could ameliorate the acute phase of cisplatin‐induced renal injury in nude rats when compared to cisplatin‐injured animals, as shown by the significant reduction in FITC‐sinistrin *t*_1/2_, sCr, serum urea and urinary albumin (Fig. 2C–2E; Supporting Information Fig. S3C). Of note, CD133^−^ cells had a similar beneficial effect on kidney function, since the differences in measurements of all four parameters between the CD133^+^ and CD133^−^‐treated animals were not significant.

### CD133^+^ and CD133^−^ Human Kidney Cells Ameliorate Histological Damage

**Figure 3:**
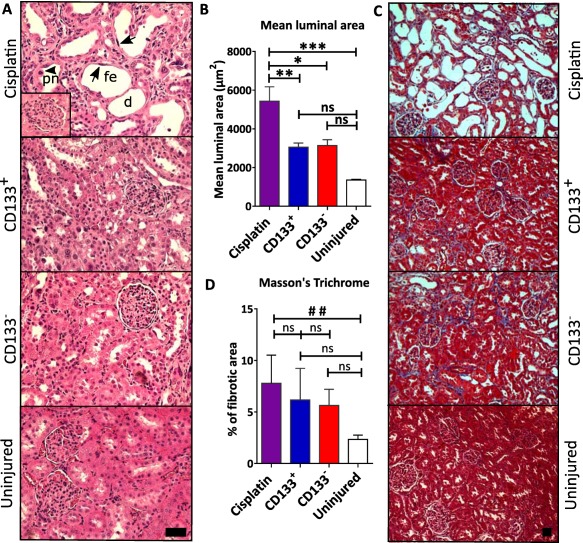
Both CD133^+^ and CD133^−^ human kidney cells ameliorate histological damage. **(A)**: Representative images of H&E‐stained kidney sections for each experimental group, including uninjured rats. The nephron tubules are noticeably damaged in the cisplatin‐injured rats that did not receive cell therapy, but the glomeruli appear normal (see inset). Magnification ×200, scale bar 50 µm. **(B)**: The mean luminal area is significantly reduced in both cell‐treated groups; **, *p* = .0084; *, *p* = .0147; ***, *p* = .0005; one‐way ANOVA statistical test with Dunnet post hoc analysis; error bars represent SEM. **(C)**: Representative images of Masson's trichrome staining of kidney sections for each experimental group, including uninjured rats. Magnification ×100, scale bar 50 µm. **(D)**: Quantification of the fibrotic area in histological kidney sections after processing of at least 10 images per animal for each group; one‐way ANOVA statistical test with Bonferroni post hoc analysis; error bars represent SEM. ##, *p* = .096. Abbreviations: d, dilated tubuli; fe, arrows—flat epithelium; ns, not significant; pn, arrowhead—pyknotic nuclei.

Next, we used histopathological analysis to investigate the impact of the administered cells on renal tissue health at day 14. In H&E‐stained sections from kidneys of the cisplatin‐injured group, abnormalities typical of acute tubular injury were observed, including cells with pyknotic nuclei and dilated tubules with flattened epithelia (Fig. 3A). By contrast, these features were rarely observed in kidneys from the cell‐treated groups and uninjured control kidneys (Fig. 3A).

We assessed the tubular damage by measuring the mean luminal area, which revealed that the tubular luminal area of cisplatin‐injured rats was significantly greater than that of the cell‐treated groups and uninjured control rats (cisplatin‐only: 5,400 ± 739 µm^2^, *n* = 7; CD133^+^: 3,000 ± 218 µm^2^ [*p* < .01], *n* = 7; CD133^−^: 3,100 ± 301 µm^2^ [*p* < .05], *n* = 6; uninjured: 1,350 ± 26 µm [*p* < .001]) (Fig. 3B).

Previous studies have shown that by 14 days following cisplatin administration, extensive fibrosis is present in rat kidneys 41. To determine whether administration of the CD133^+^ and CD133^−^ cells could ameliorate renal fibrosis, we performed MT analysis. Despite widespread tubular dilation in the cisplatin‐injured group, fibrotic lesions were minimal and no significant differences were observed between the cisplatin‐injured and cell‐treated groups at day 14 (Fig. 3C, 3D; Supporting Information Fig. S4). The negligible degree of renal fibrosis in these animals is likely due to the fact that they lack T cells, which are known to play a key role in the development of renal fibrosis 42.

### CD133^+^ and CD133^−^ Cells do not Engraft in the Kidneys Following IV Administration

Previous studies have suggested that intravenously administered CD133^+^ human kidney cells ameliorate injury by engrafting in the kidney and generating podocytes and PTCs 17, 19. MSCs, on the other hand, become entrapped in the lung following IV administration and ameliorate renal injury via paracrine or endocrine factors 12, 14, 43. To investigate whether CD133^+^ and CD133^−^ human kidney cells were present in the kidneys or lungs following IV administration in the rat cisplatin model, tissue sections were analyzed for the presence of GFP and PKH26 at day 14.

**Figure 4:**
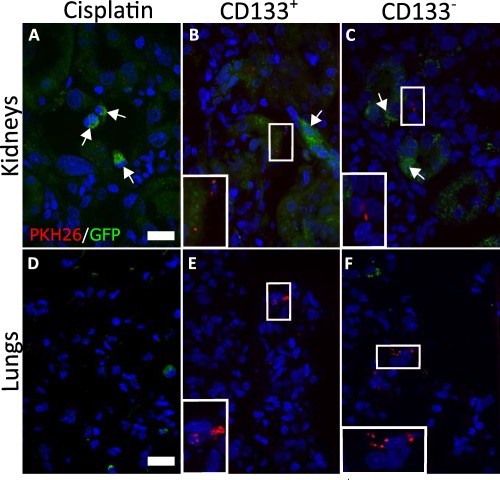
PKH26 dye but not GFP‐positive cells are found in kidneys and lungs. Maximum intensity projection (MIP) confocal microscopy images of representative tissue sections of kidneys **(A–C)** and lungs **(D–F)**. In the kidneys, green autofluorescence is observed in all groups (white arrows). No GFP^+^ cells could be detected in either the kidneys or the lungs. Punctate PKH26 label was found in both organs. Magnification: ×400, scale bars 100 µm. Abbreviations: GFP, green fluorescent protein.

PKH26 could be detected in the kidneys of rats that received human kidney cells, and was typically located close to tubular or interstitial cells, but there was no evidence of any GFP^+^ cells (Fig. 4B, 4C). Similarly, no GFP^+^ cells, but traces of PKH26 dye, were identified in the lungs of animals treated with human kidney cells (Fig. 4E, 4F). As expected, neither PKH26 nor GFP^+^ cells were found in kidneys or lungs of cisplatin‐injured animals that did not receive cells (Fig. 4A, 4D, see Supporting Information Fig. S5 for a comparison between a GFP^+^ cell and an autofluorescent cell). The presence of PKH26 in the absence of GFP suggested that PKH26 staining at day 14 was due to false positive staining of host cells. Overall, these results indicated that the administered cells were not detectable in kidneys or lungs by day 14.

**Figure 5:**
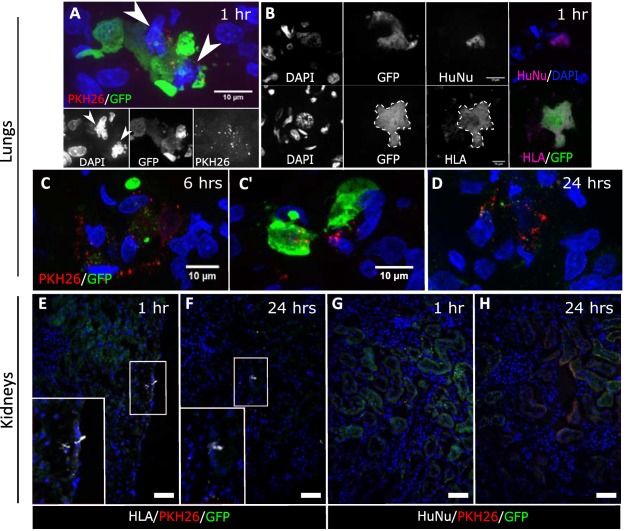
Human kidney cells are entrapped in the lungs and die within 24 hours. **(A, B)**: Representative MIP confocal microscopy images of lung sections of animals at 1, 6, and 24 hours following cell administration. One hour after injection, some GFP^+^ cells had apoptotic nuclei, indicated by the arrowheads (A). (B) To confirm the identity of the GFP+ cells, immunostaining was performed with the human specific antibodies, HLA and human nuclei‐HuNu. **(C, C′)**: By 6 hours after injection, a few intact GFP^+^ cells were still present, and PKH26 was observed in neighboring non‐GFP host cells. **(D)**: By 24 hours, PKH26 was found in a punctate pattern, near GFP‐labeled cell fragments. **(E–H)**: Representative confocal microscopy images showing the kidney cortex of animals sacrificed at 1 and 24 hours following cell administration, stained for HLA (E, F) or HuNu (G, H) and GFP. No GFP^+^ cells were detected in the kidneys at any time point. Magnification: (A–D) ×1000, (E–H) ×200, scale bars 50 µm unless specified. Abbreviations: GFP, green fluorescent protein; DAPI, 4′,6‐diamidino‐2‐phenylindole; HLA, human leukocyte antigen; HuNu, human nuclei.

To demonstrate the presence of the human kidney cells and investigate their fate at earlier time points, CD133^+^ human kidney cells were IV injected into cisplatin‐injured rats and tissue sections analyzed for the presence of cells at 1, 6, and 24 hours after cell administration. Using GFP‐ and human‐specific antibodies (i.e., against HLA and a human nuclear antigen), we could detect GFP^+^ human cells in sections of lungs collected one hour after cell administration (Fig. 5A, 5B). However, the presence of pyknotic nuclei and blebbing in some of the GFP^+^ cells suggested that they were undergoing cell death. In addition, we could identify a punctate pattern of PKH26 dye in or around the cells. In sections of lungs collected six hours after cell administration, we found both fragmented and intact GFP^+^ cells. Importantly, we detected the PKH26 label not only in GFP^+^ cells, but also in neighboring GFP^−^ cells (Fig. 5C, 5C′). At the 24‐hour time point, we could only identify the GFP signal in cell fragments close to PKH26^+^ puncta, but not in intact cells (Fig. 5D). By contrast, analysis of kidney sections at all three time points revealed only punctate PKH26 staining, but no GFP^+^ cells or fragments. HLA staining was occasionally detected in the kidney, but no human nuclei were observed, confirming the lack of engraftment of intact cells in this organ (Fig. 5E–5H). To investigate whether the cells had engrafted in the spleen and liver, tissue sections from these organs were stained for GFP and the human‐specific antigens, but only background autofluorescence could be detected (see Supporting Information Fig. S6 for spleen data; liver data not shown). Therefore, our data demonstrate that following tail vein injection, the human kidney cells became entrapped in the lungs and died within 24 hours of administration. Importantly, our results provide no evidence of the cells engrafting in the kidneys.

### IV Injection of Human Kidney Cells Leads to Macrophage Infiltration in the Lungs

**Figure 6:**
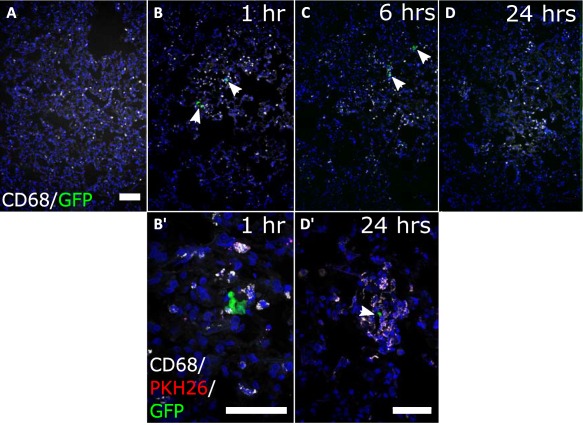
CD68^+^ phagocytic cells cluster around the human kidney cells in the lungs. **(A–D)**: Representative maximum intensity projection (MIP) confocal microscopy images showed CD68 signal (white) in lung sections from control and cell‐administered animals at all time points. In sections from animals that received human kidney cells, CD68^+^ cells were clustered around the GFP‐labeled cells (white arrows). Scale bar is 100 µm. **(B′, D′)**: MIP confocal microscopy images at higher magnification, showing GFP (green), PKH26 (red), and CD68 (white). One hour after administration, CD68^+^ cells were found clustered around the human cells in sections of lungs. (D′) By 24 hours, some CD68^+^ cells were localized around GFP^+^ cell fragments (white arrow). Magnification: (A–D) ×100; (B′–D′) ×400, scale bars 50 µm. Abbreviation: GFP, green fluorescent protein

Dying cells and their fragments are known to be phagocytosed by resident macrophages 44 and dendritic cells 45. Furthermore, the process of macrophage‐based removal of apoptotic cells has been shown to inhibit inflammation via anti‐inflammatory cytokines 46, 47. To determine whether macrophages were recruited to the dying human cells, we performed immunofluorescence staining for the pan‐macrophage marker CD68 on sections from lungs at 1, 6, and 24 hours after administration of human kidney cells. We found that the CD68^+^ cells were evenly distributed across the tissue in sections of control lungs from all time points (Fig. 6A). By contrast, at one and 6 hours after cell administration, CD68^+^ cells appeared to cluster closely around intact GFP^+^ cells and GFP^+^ cell fragments (Fig. 6B, 6B′, 7C). Confocal imaging and volume rendering of z‐stacks revealed that some of the CD68^+^ cells also contained the PKH26 label (see Supporting Information Videos 1 and 2), suggesting that the macrophages might be involved in phagocytosing the GFP^+^ CD133^+^ cell fragments. In sections of lungs at 24 hours after cell administration, the CD68^+^ cells remained in clusters around small GFP‐labeled fragments while intact human cells were no longer present (Fig. 6D, 6D′). Analysis of expression of the anti‐inflammatory cytokine IL10 in the lungs at 24 hours showed IL10 within and surrounding CD68^+^ cells in close proximity to the dying human cells. These results suggest that IL10 may have been released by the macrophages following phagocytosis of the human cells (Supporting Information Fig. S7).

## Discussion

In this study, we have shown that CD133^+^ cells isolated from human infant renal tissue could improve renal function and ameliorate tissue damage following IV administration in a rat cisplatin model. These findings are consistent with previous studies that showed adult kidney‐derived CD133^+^ cells had beneficial effects following tail vein injection in mouse models of glomerular and tubular injury 19, 48. However, whereas these earlier studies reported no beneficial effects of CD133^−^ cells, we found that in the rat cisplatin model, CD133^+^ and CD133^−^ cells were similarly therapeutic (Table 1).

**Table 1:** Summary of the therapeutic efficacy observed in both cell‐treated groups compared with the control groups

	CD133^+^	CD133^−^
FITC‐Sinistrin *t*_1/2_	↓59.1%	↓46.8%
sCreatinine	↓72.3%	↓64.7%
sUrea	↓74.2%	↓63.6%
albumin:creatinine ratio	↓90.9%	↓90.3%
Histological damage	↓43.8%	↓42.2%
Fibrosis	N/A	N/A

Percentage changes apply for FITC‐sinistrin *t*_1/2_, serum creatinine, and BUN at day 7, and for albumin:creatinine ratio, histological damage, and fibrosis at day 14

Abbreviation: FITC, fluorescein isothiocyanate.

CD133 has previously been identified as a marker for various types of stem cells 18, and has been reported to be a progenitor cell marker in human fetal 49 and adult kidneys 16, 17, 19. In the fetal kidney, this was mainly based on evidence that CD133 was expressed in the metanephric mesenchyme 49, a population of cells within the developing mammalian kidney that gives rise to all cell types of the nephron. However, more recent analysis has shown that CD133 is not coexpressed with the bona fide nephron progenitor marker, SIX2, and is instead expressed in differentiating cells 50, suggesting that CD133 is not a progenitor marker in the fetal kidney. In adult human kidneys, CD133 is expressed in parietal epithelial cells 17 and in a small population of scattered tubular epithelial cells within the proximal and distal tubules 17, 51, 52, which is consistent with the staining pattern observed in infant kidneys in the current study. The most compelling evidence for CD133 being a renal progenitor marker is based on studies showing that following IV administration into mice with renal injury, adult human kidney‐derived CD133^+^ cells could engraft in the kidneys and generate podocyte and PTCs 17, 19. However, this contrasts with our current study, where we found that following tail vein injection, infant kidney‐derived CD133^+^ and CD133^−^cells did not engraft into injured kidneys, and instead, were entrapped in the lungs and did not survive beyond 24 hours.

A possible explanation for these apparent differences in CD133^+^ biodistribution could be due to the fact that in the earlier studies, CD133^+^ cells were isolated from the “normal” regions of adult kidneys affected by tumors. This raises the possibility that the adult CD133^+^ cells might have been renal carcinoma cells that had migrated from the primary lesion into the surrounding normal tissue, and perhaps as a result of their malignant phenotype, were able to traverse the pulmonary capillaries. It must also be considered that in the earlier studies, the cells were used at passage 0 (P0), whereas in the current study, the cells were used at passage 5 (P5). Therefore, we cannot discount the possibility that P0 cells might be able to pass through the lung, whereas P5 cells cannot. However, it is more likely that the high level of renal engraftment reported in these previous studies was due to a combination of autofluorescence 53 and false‐positive staining, for it is known that the lipophilic dye, PKH26, that was used to monitor the fate of the CD133^+^ cells, can readily transfer to host cells 22, 43. Indeed, the problem with PKH26 false‐positive staining was demonstrated in the current study, where we detected PKH26 dye in the kidneys despite the GFP‐labeled CD133^+^ cells being located solely in the lungs. It is also worth noting that the high levels of renal engraftment reported in some earlier studies would be difficult to achieve (see Supporting Information Table S2) 17.

The lack of renal engraftment in the current study indicates that the therapeutic effects of the CD133^+^ and CD133^−^ cells in the rat cisplatin model are mediated by paracrine or endocrine factors. Our results are similar to those of a previous study by Geng and colleagues 14 investigating the therapeutic effects of MSCs in a mouse tubular injury model, where it was found that following tail vein administration, cells were mainly located in the lungs and were not detected in the kidneys. Further experiments showed that the MSCs ameliorated renal injury by increasing the number of alternatively‐activated (M2) macrophages 14. It is well‐established that MSCs have immunomodulatory effects and ameliorate renal injury by paracrine mechanisms 12, 13, but more recently, it has been shown that the therapeutic effects of various other cell types, including human induced pluripotent stem cell (iPSC)‐derived nephron progenitors, are also mediated by paracrine factors 54. For instance, Toyohara et al. showed that if iPSC‐derived nephron progenitors were injected into the renal parenchyma of mice with ischemia reperfusion injury, the cells could integrate and generate new tubular cells, but did not ameliorate injury 54. On the other hand, if the cells were injected under the kidney capsule, they did not integrate into the kidney, but had a significant therapeutic effect. Likewise, the administration of rat kidney‐derived renal tubule cells into rats with subtotal nephrectomy reduced leucocyte infiltration and inflammation, and promoted repair via paracrine mechanisms 55. Similar findings have been reported for other organs, such as the liver and heart, where it was respectively shown that the therapeutic effects of embryonic stem cell‐derived hepatocytes and cardiac stem cells were mediated by trophic factors 56, 57.

Our observation that the CD133^+^ and CD133^−^ cells had similar therapeutic effects in the rat cisplatin model is interesting, especially given the differences in phenotype between the two cell types, with the former being predominantly epithelial cells, and the latter having a more mesenchymal‐like morphology. An important finding from our study was that similarly to MSCs 58, the human kidney cells died quite rapidly in the lung following IV administration and induced the infiltrating macrophages to express IL10. It is well established that apoptotic cells release “find me” signals, such as fractalkine and lysophosphatidylcholine, which serve as chemo‐attractants for macrophages 45. Moreover, following the phagocytosis of apoptotic cell debris, macrophages become polarized toward an alternatively activated (M2) phenotype and secrete anti‐inflammatory cytokines, such as IL10 59, which has been shown to ameliorate cisplatin‐induced renal injury in rodents 60. Thus, it is possible that the therapeutic effects of the dying human kidney cells might have been due to their ability to trigger macrophages to secrete IL10 (and perhaps other anti‐inflammatory cytokines), which could stimulate renal repair by promoting an early resolution of the inflammatory response. In support of a role for dying cells, Thum et al. 46 have previously hypothesized that immunomodulatory effects can be triggered by the apoptosis of exogenous stem cells, and more recently, Luk et al. have shown that heat‐inactivated (i.e., nonviable) MSCs can modulate macrophages and induce a dramatic increase in IL10 expression in mice 61.

Furthermore, dying (apoptotic) cells have also been reported to release microvesicles or apoptotic bodies which could potentially have immunomodulatory roles in kidney regeneration 62. This is supported by the observation that microvesicles isolated from MSCs have renoprotective roles in mouse models of AKI 63, 64, 65.

Though beyond the scope of the present study, by understanding the mechanisms whereby dying cells modulate the behavior of key immune effector cells and ameliorate renal injury, it should be possible to develop safer and more effective cell‐free regenerative medicine therapies in the future.

## Conclusion

Here we present data showing that cells isolated from human infant kidneys have the potential to ameliorate functional and histological damage in rats with cisplatin‐induced kidney injury. Our results demonstrate that CD133 expression in the cells is not required for their beneficial effects. Furthermore, since the human kidney cells die in the lungs shortly after their administration and are not detected in the kidneys, our findings indicate that the cells exert their therapeutic effects via paracrine factors, and that renal engraftment is not a prerequisite for ameliorating renal injury.

## Author Contributions

I.S.: conception and design, collection and/or assembly of data, data analysis and interpretation, manuscript writing, final approval of manuscript; Z.H.P.: conception and design, collection and/or assembly of data, data analysis and interpretation, final approval of manuscript; A.T. and A.I.: collection and/or assembly of data, final approval of manuscript; S.K.: provision of study material; N.G., P.M., and B.W.: conception and design, financial support, data analysis and interpretation, manuscript writing, final approval of manuscript.

## Disclosure of Potential Conflicts of Interest

S.K. Consultant Paediatric Surgeon, Alder Hey Children's Hospital NHS Foundation Trust. The other authors indicated no potential conflicts of interest.

## Supporting information

Supporting Information Figure 1.

Supporting Information Figure 2.

Supporting Information Figure 3.

Supporting Information Figure 4.

Supporting Information Figure 5.

Supporting Information Figure 6.

Supporting Information Figure 7.

Supporting Information Table 1.

Supporting Information Table 2.

Supporting Information Video 1.

Supporting Information Video 2.

Supporting Information.
