# Supplementary figures and images for: Human Kidney‐Derived Cells Ameliorate Acute Kidney Injury Without Engrafting into Renal Tissue
Source: Stem Cells Transl Med. 2017 Apr 4;6(5):1373–84. doi: 10.1002/sctm.16-0352 (PMC5442715; doi:10.1002/sctm.16-0352)

# Supplemental Figure 1

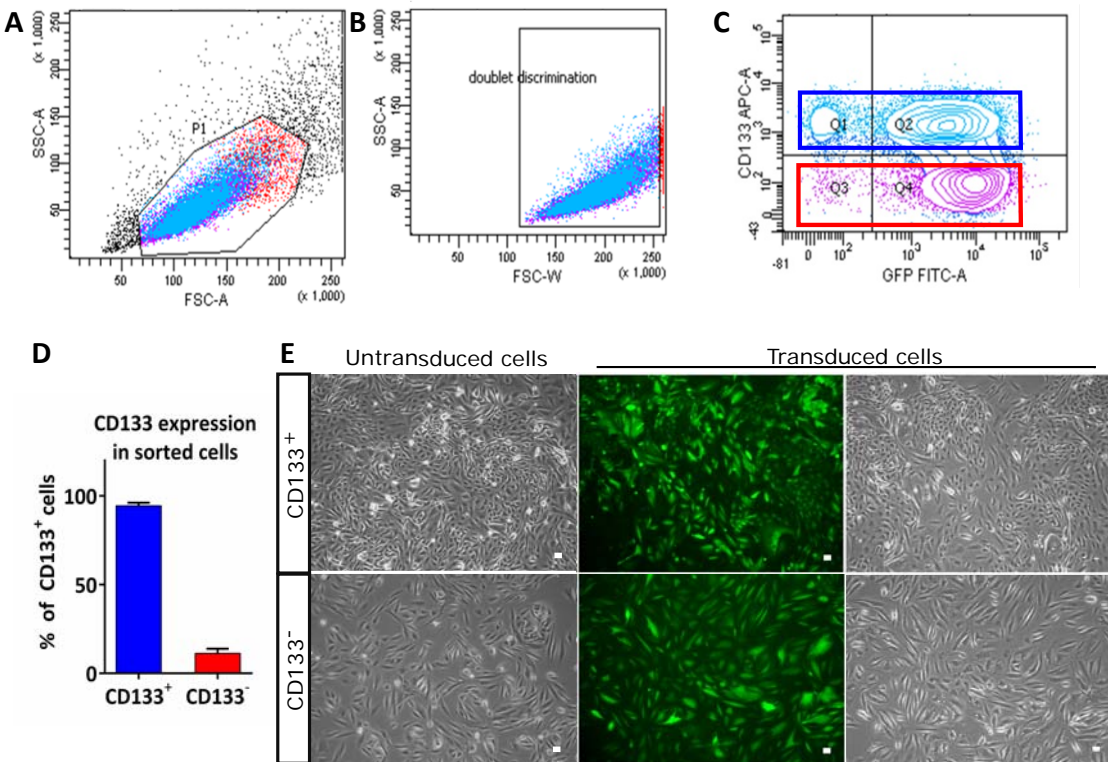

Supplement: Supplementary file 1 — Supporting Information Figure 1. [file SCT3-6-1373-s001.pdf]

# Supplemental figure 3

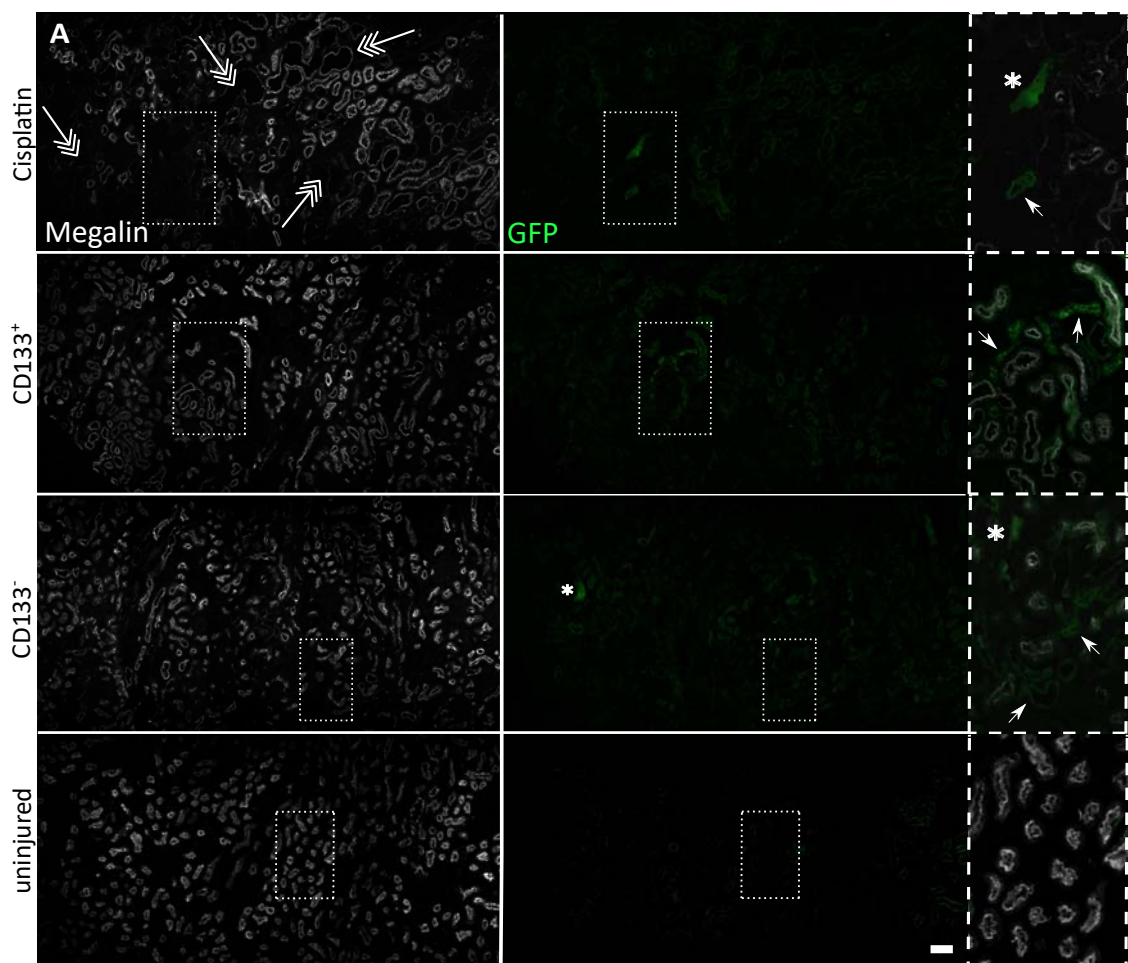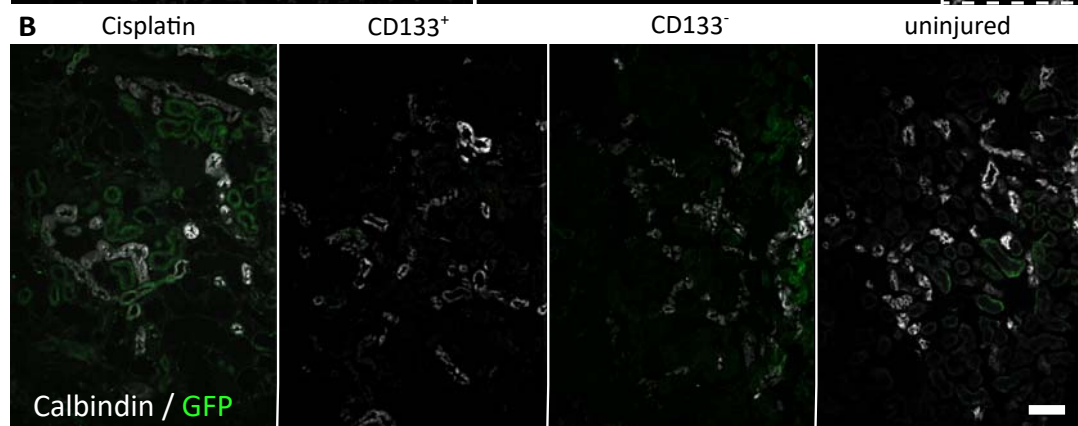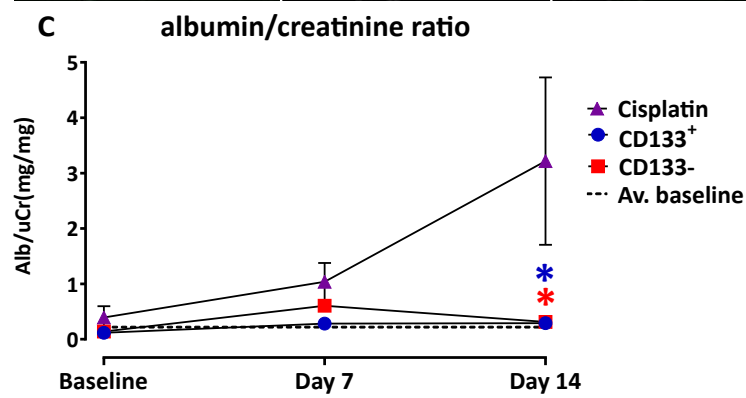

Supplement: Supplementary file 3 — Supporting Information Figure 3. [file SCT3-6-1373-s003.pdf]

Supplemental Figure 4

Cisplatin

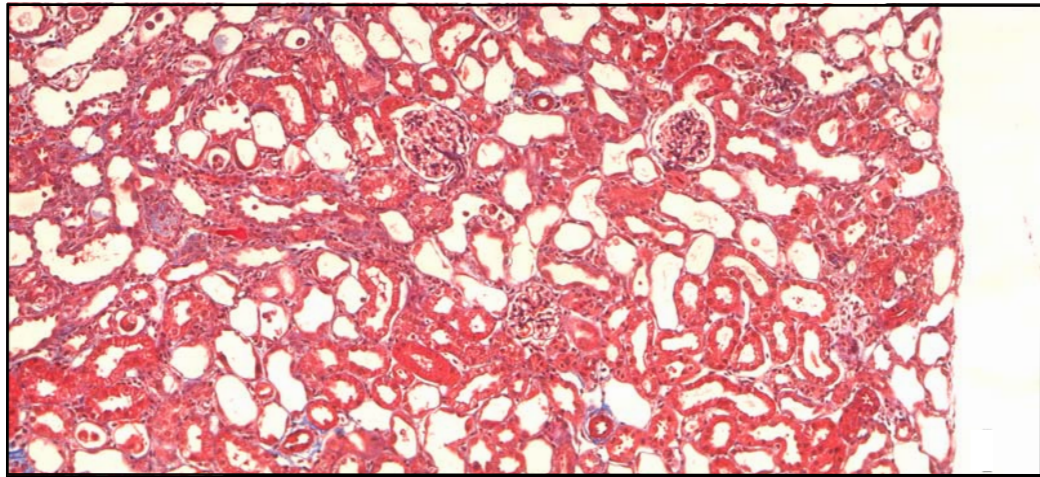

CD133<sup>+</sup>

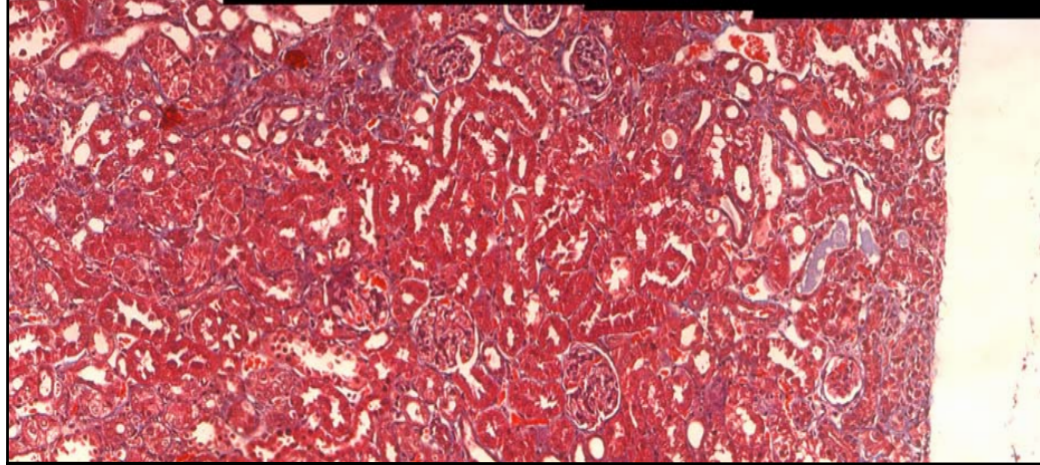

CD133<sup>-</sup>

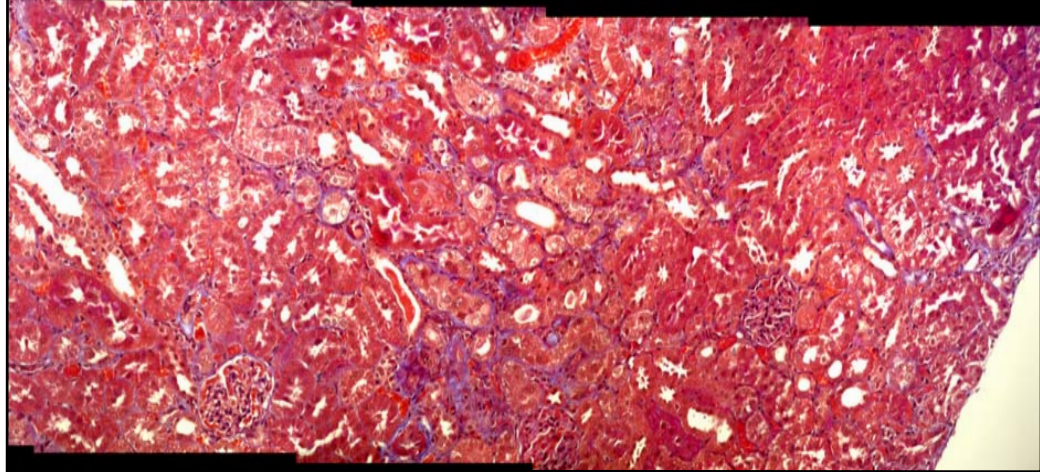

Uninjured

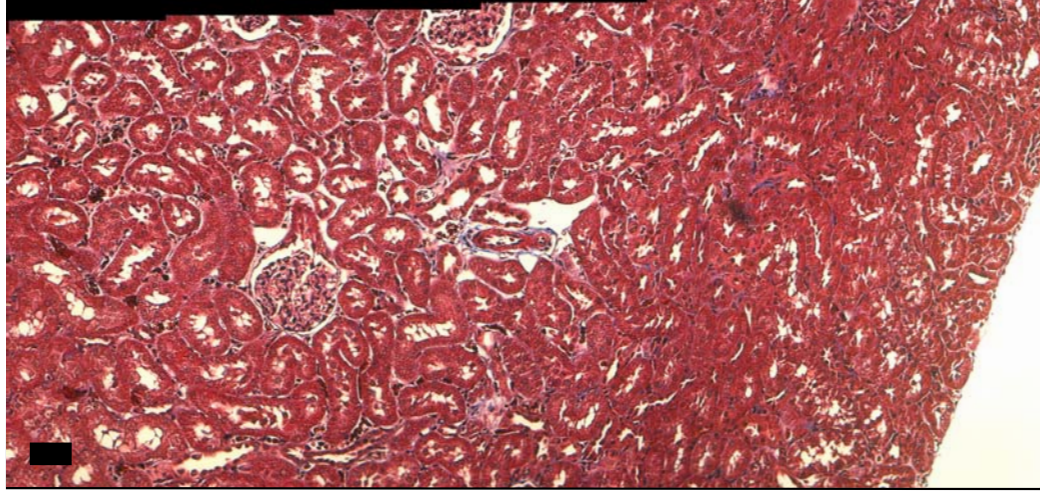

Supplement: Supplementary file 4 — Supporting Information Figure 4. [file SCT3-6-1373-s004.pdf]
