# Supplementary material for: Human Kidney‐Derived Cells Ameliorate Acute Kidney Injury Without Engrafting into Renal Tissue
Source: Stem Cells Transl Med. 2017 Apr 4;6(5):1373–84. doi: 10.1002/sctm.16-0352 (PMC5442715; doi:10.1002/sctm.16-0352)
Supplement: Supplementary file 2 — Supporting Information Figure 2. [file SCT3-6-1373-s002.pdf]

Supplemental Figure 2

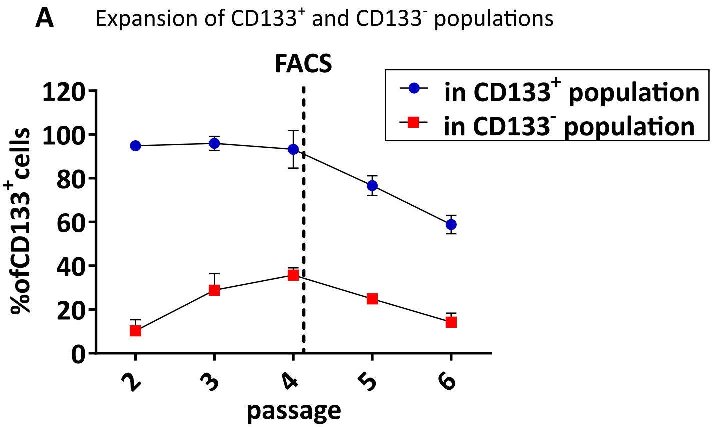

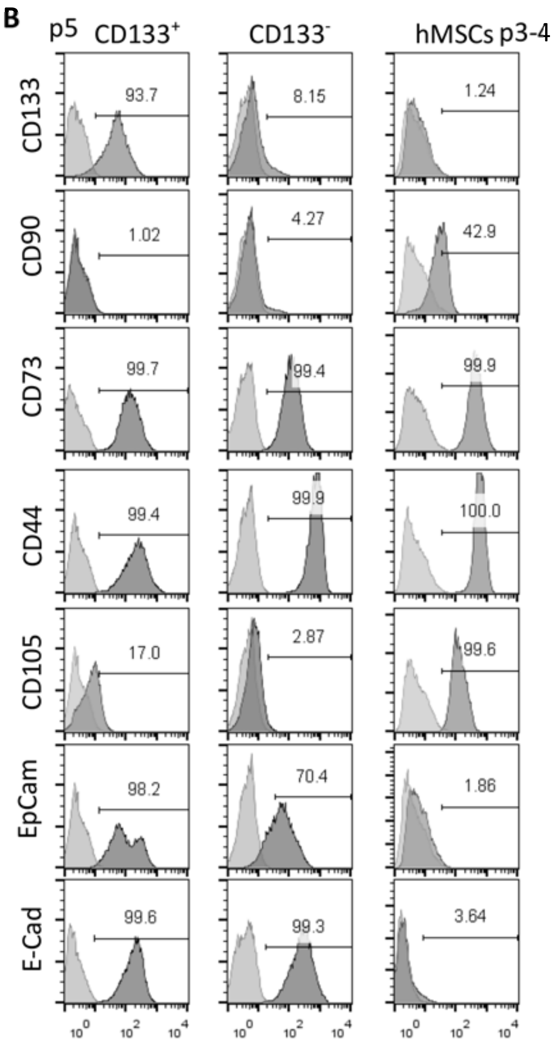

C

Marker	CD133$^+$	CD133$^-$	hMSCs
CD133	89 ± 6.65	11.63 ± 4.91	0.98 ± 0.19
CD90	1.175 ± 0.52	10.91 ± 9.46	14.41 ± 18.35
CD73	99.8 ± 0.14	99.35 ± 0.49	97.56 ± 2.71
CD44	99.65 ± 0.35	98.65 ± 1.63	98.73 ± 1.25
CD105	12.425 ± 6.61	1.97 ± 1.33	89.23 ± 9.3
CD326	98.85 ± 1.34	67.95 ± 3.46	1.24 ± 0.53
CD324	99.7 ± 0.14	99.3 ± 0.14	3.42 ± 1.63
