# Supplementary material for: Human Kidney‐Derived Cells Ameliorate Acute Kidney Injury Without Engrafting into Renal Tissue
Source: Stem Cells Transl Med. 2017 Apr 4;6(5):1373–84. doi: 10.1002/sctm.16-0352 (PMC5442715; doi:10.1002/sctm.16-0352)
Supplement: Supplementary file 8 — Supporting Information Table 1. [file SCT3-6-1373-s008.docx]

**Supplemental Table 1**

**ns**

******

*******

***

|  |  | *Cisplatin* |  | *CD133^+^* |  | *CD133^-^* |
| --- | --- | --- | --- | --- | --- | --- |
| FITC-sinistrin t_1/2_ (min)  ± SEM | ***baseline*** | 16.41 ± 1.78 |  | 17.36 ± 1.54 |  | 13.38 ± 1.68 |
|  | ***d2*** | 40.3 ±12.51 |  | 30.63 ± 2.08 |  | 27.25 ± 2.07 |
|  | ***d7*** | 81.27 ± 11.01 |  | 33.21 ± 4.47 |  | 43.18 ± 4.96  ***** |
|  | ***d14*** | 50.83 ± 7.47 | ******* | 35.07 ± 5.38 |  | 37.60 ± 5.17 |
| sCr (mg/dL)  ± SEM | ***baseline*** | 0.16 ± 0.00 |  | 0.17 ± 0.00 |  | 0.16 ± 0.00 |
|  | ***d7*** | 1.59 ± 0.55 |  | 0.44 ± 0.04 |  | 0.56 ± 0.05 |
|  | ***d14*** | 0.59 ± 0.08 |  | 0.39 ± 0.04 | ******* | 0.46 ± 0.02 |
| Urea (mg/dL) ± SEM | ***baseline*** | 39.64 ± 2.09 | ******* | 31.8 ± 1.33 |  | 35.23 ± 1.80 |
|  | ***d7*** | 294.7 ± 94.87 |  | 75.84 ± 10.97 |  | 107.3 ± 24.82 |
|  | ***d14*** | 122.1 ± 29.23 | ******* | 63.7 ± 10.4 |  | 76.42 ± 12.32  **ns** |
