# Supplementary material for: Human Kidney‐Derived Cells Ameliorate Acute Kidney Injury Without Engrafting into Renal Tissue
Source: Stem Cells Transl Med. 2017 Apr 4;6(5):1373–84. doi: 10.1002/sctm.16-0352 (PMC5442715; doi:10.1002/sctm.16-0352)
Supplement: Supplementary file 9 — Supporting Information Table 2. [file SCT3-6-1373-s009.docx]

**Supplemental table 2.** Estimation of the extent of renal engraftment of CD133+ cells reported in a previous study^a1^

|  | **Approximate number of podocytes and proximal tubule cells in an adult mouse** | | | | | | |
| --- | --- | --- | --- | --- | --- | --- | --- |
|  | podocytes/ glomerulus^2^  **A** | Average length PT^3^  **B** | Length of PTC basal surface^4^  **C** | Cells per PT cross-section^4^  **D** | Nephrons per kidney^5^  **E** | Total cells/ kidney  **F** | Total cells/ mouse  **G** |
| Total no. podocytes | 90 |  |  |  | 2,000 | A x E  180,000 | F x 2  360,000 |
| Total no. PTCs |  | 4000 µm | 10 µm | 8 | 2,000 | B/C X D x E  6,400,000 | F x 2  12,800,000 |
|  | **Extent of CD133+ cell engraftment reported in previous study^1^** | | | | | | |
|  | No. cells injected  **H** | No. occasions  **I** | % engraftment  **J** | No. cells engrafting  **K** | Total no. engrafting cells  **L** | % injected dose engrafting | Maximum % cells expected to engraft |
| Glomerular engraftment | 750,000 | 2 | 11% | J X G  ~40,000 | 940,000 | L/(H X I) X 100  63% | 20% |
| PT engraftment |  |  | 7% | J X G  ~900,000 |  |  |  |

1. Ronconi E, Sagrinati C, Angelotti ML, et al. Regeneration of glomerular podocytes by human renal progenitors. *Journal of the American Society of Nephrology : JASN.* 2009;20(2):322-332.

2. Nicholas SB, Basgen JM, Sinha S. Using stereologic techniques for podocyte counting in the mouse: shifting the paradigm. *Am J Nephrol.* 2011;33 Suppl 1:1-7.

3. Zhai XY, Birn H, Jensen KB, Thomsen JS, Andreasen A, Christensen EI. Digital three-dimensional reconstruction and ultrastructure of the mouse proximal tubule. *Journal of the American Society of Nephrology : JASN.* 2003;14(3):611-619.

4. Sakamoto H, Sado Y, Naito I, et al. Cellular and subcellular immunolocalization of ClC-5 channel in mouse kidney: colocalization with H+-ATPase. *Am J Physiol.* 1999;277(6 Pt 2):F957-965.

5. Murawski IJ, Maina RW, Gupta IR. The relationship between nephron number, kidney size and body weight in two inbred mouse strains. *Organogenesis.* 2010;6(3):189-194.
