# Supplementary material for: Human Kidney‐Derived Cells Ameliorate Acute Kidney Injury Without Engrafting into Renal Tissue
Source: Stem Cells Transl Med. 2017 Apr 4;6(5):1373–84. doi: 10.1002/sctm.16-0352 (PMC5442715; doi:10.1002/sctm.16-0352)
Supplement: Supplementary file 12 — Supporting Information. [file SCT3-6-1373-s012.docx]

# Human kidney-derived cells ameliorate acute kidney injury without engrafting into renal tissue

Running title: human kidney cells improve AKI without engrafting

Ilaria Santeramo^1^*, Zeneida Herrera Perez^2^*, Ana Illera^1^, Arthur Taylor^1^, Simon Kenny^3^, Patricia Murray^1§^, Bettina Wilm^1§^, Norbert Gretz^2§^

# Supplemental Data

## Supplemental methods

## Human cells and tissue immunofluorescence

Upon reception, part of the human tissue was frozen for immunhistological analysis. 6 µm-thick sections were blocked with 0.1% Triton-X 100 (Sigma) and 10% Goat Serum (Sigma) in PBS and incubated with anti-CD133/1 (1: 50, Miltenyi) followed by secondary antibody Alexa Fluor^®^ 488 nm goat anti-mouse IgG_1_ (Thermo-Fisher Scientific). Fluorescence images were taken using a spinning disk confocal microscope CSU-X1 (3i), coupled with a digital camera (CMOS, Hamamatsu).

For the immunofluorescence experiments with cells, freshly isolated cells were plated in 8-well chamber slides (Corning), and grown until confluent. The cells were fixed in 4% PFA, blocked using 2% BSA (Sigma), 0.1% Triton-X in PBS and incubated with anti-CD133/2 (1:50, Miltenyi) followed by secondary antibody Alexa Fluor^®^ 594-coupled goat anti-mouse IgG (H+L) (Thermo-Fisher Scientific). Fluorescence images were taken using an epifluorescence Leica DM2500 microscope coupled to a Leica DFC420C camera.

## Flow cytometry and FACS

For the flow cytometry analysis of the CD133^+^ and CD133^-^ (shown in Figure 1C) cells, a BD FACS Calibur (BD Biosciences) was used. 5x10^5^ cells were labelled with CD133/1 APC (Miltenyi, 1:11), CD24 FITC (Miltenyi, 1:11), or both in 1% (v/v) FBS, 0.1% NaN_3_ in PBS, according to the manufacturer instructions. Unlabelled cells were used to set the FSC, SSC and the fluorescence channels correctly. For FACS, a BD FACS Aria (BD Biosciences) was used. Cells were labelled with CD133/1 APC (1:11) in 1% (v/v) FBS at a concentration of 5-7.5x10^6^ cells/ml. 5x10^5^ cells unlabelled or labelled cells were used to set the gates. Following the sorting, both populations were re-analysed to define the purity of the sorting and plated.

## Expansion in culture of CD133^+^ and CD133^-^ cells and flow cytometric analysis

CD133^+^ and CD133^-^ cells were isolated using flow activated cell sorting (FACS) and expanded in culture. A low plating density was used for both cell types (2 x 10^5^ per 10cm^2^ plate). The expression of CD133/1 APC was analysed by flow cytometry at each passage, using a BD FACS Calibur (BD Biosciences). In order to maintain a pure population of CD133^-^ cells, magnetic activated cell sorting (MACS) was performing using CD133/1 PE (Miltenyi, 1:11) and Anti-PE Microbeads (Miltenyi, 40µl on LD columns). At passage 5 a sample of the CD133^+^ and CD133^-^ cells that were administered to the animals were analysed using flow cytometry. 0.5-1 x 10^6^ cells were labelled with CD133/1 APC, CD133/2 PE, CD24 FITC, CD90 FITC, CD73 PE, CD105 FITC, CD44 FITC, CD326 FITC, CD324 APC (all from Miltenyi, 1:11) in 1% (v/v) FBS, 0.1% NaN_3_ in PBS, according to the manufacturer’s instructions. Unlabelled cells were used to set the gates for the forward scatter (FSC) and side scatter (SSC) and isotype specific control antibodies were used to set the gates for the fluorescence channels. For analysis of CD133^+^ and CD133^-^ cells, antibodies were used in combination with CD133/1 or CD133/2. Human MSCs were from Lonza and cultured according to the manufacturer’s recommendations. 0.5-1 x 10^6^ cells at passage 3 or 4 were labelled with CD133/1 APC, CD133/2 PE, CD24 FITC, CD90 FITC, CD73 PE, CD105 FITC, CD44 FITC, CD326 FITC, CD324 APC (all from Miltenyi, 1:11) in 1% (v/v) FBS, 0.1% NaN_3_ in PBS, according to the manufacturer’s instructions.

# Supplemental Figures

**Supplemental Figure 1.** Sorting for GFP^+^ CD133^+^ and GFP^+^ CD133^-^ kidney cells. **(A)** Dot Plot showing FSC-A (Forward Scatter-Area) and SSC-A (Side Scatter-Area) used to discriminate healthy and damaged or dying cells in the bulk population. **(B)** Dot plot of SSC-A and FSC-W (Forward Scatter-Width) used to discriminate doublets, which might represent a false positive signal. **(C)** Density plot showing the signal from the excitation of cells with the 488-nm laser (GFP, FITC-A, log scale) against the signal from the FL4 laser (CD133, APC-A, log scale). A small percentage of CD133^+^ cells was not efficiently transduced with GFP lentivirus (CD133^+^ 4.9% vs CD133^-^ 1.4%). **(D)** Percentage of CD133-expressing cells after sorting into CD133^+^ and CD133^-^ populations. **(E)** Representative images of transduced and un-transduced CD133^+^ cells and CD133^-^ cells at passage 3. The CD133^+^ population mainly comprised epithelial cells, while the CD133^-^ population contained mostly elongated mesenchymal-like cells. Magnification 100x, scale bars 100 µm.

**Supplemental Figure 2. (A)** Flow cytometric analysis performed on CD133^+^ and CD133^-^ cells from passage 2 (P2) to passage 6 (P6). In order to achieve a high purity of the CD133^-^ population, CD133-specific MACS was used to purify the population at P3 and P4. The flow cytometric analysis of the cells at P5 (time-point of injection) is reported in **(B).** Characterization of the CD133^+^_,_ CD133^-^ (P5, n=2) and human MSCs (hMSCs, P3-4, n=3) for mesenchymal and epithelial markers using flow cytometry. **(C)** Table indicating average ± SD of all the markers analysed in each cell type.

**Supplemental Figure 3**. Absence of GFP+ cells and evidence of tubular regeneration in the renal cortex 14 days after cell administration. **(A)** Representative stitched confocal images of the renal cortex of animals belonging to the four experimental groups. Kidney sections were stained for megalin and GFP, and the confocal images stitched to a length of 2mm. In the cisplatin-injured group the pattern of megalin staining in some proximal tubules was atypical (arrows with triple heads), likely reflecting the fact that the PTCs in these tubules were injured. In contrast, the sections from cell-treated rats were indistinguishable from the uninjured animals. While no GFP signal was detected, unspecific green fluorescence was present, which was particularly strong in protein casts (*) and in cross-sections of tubuli (white arrows). **(B)** Representative confocal images of the renal cortex of animals stained for calbindin showed no differences between the different groups. **(C)** Albumin:creatinine ratio. The dotted line represents the average of the albumin:creatinine baseline values of all animals (n=20). An ANOVA one-way statistical test with Bonferroni’s *Post Hoc* analysis was applied to each data set to compare the groups at day 14. CD133^+^ group (n=7); CD133^-^ group (n=6); control group (n=7). * p=0.0295; * p=0.0498 Magnification **(A)** 100x **(B)** 200x, scale bars 100 µm.

**Supplemental Figure 4.** Masson’s trichrome staining shows negligible levels of fibrosis in cell-treated animals and injured controls. Representative stitched images of Masson’s trichrome staining of kidney sections from animals of cisplatin-injured group, CD133^+^-treated group, CD133^-^-treated group and uninjured group. Magnification 100x, scale bar 100μm.

**Supplemental Figure 5.** Representative confocal MIP of the lungs of an animal sacrificed 1 hour after injection showing a GFP^+^ cell (left side) and an autofluorescent cell (right). Magnification 630x, scale bars 10 μm.

**Supplemental Figure 6.** Representative confocal images of the spleen of an uninjured rat (A), cisplatin-injured rat sacrificed 1 hour after injection of PBS (B), cisplatin-injured rat sacrificed 1 hour after injection of CD133^+^ cells (C), stained for GFP (green) and HuNu (white). Background autofluorescence was present in all samples but no signal was detected for human nuclei. Magnification 400x, scale bar 50μm.

**Supplemental Figure 7.** Representative confocal MIP of the lungs of animals sacrificed 1 **(B)** and 24 **(C)** hours after injection of CD133^+^ cells stained for IL10 (red), CD68 (white), and GFP (green). At 24 hours, clusters of CD68^+^ were observed, with an increase in the staining for IL10. White arrows identify green fragments belonging to GFP^+^ cells. Magnification 400x, scale bar 50 μm.

# Supplemental Tables

**Supplemental table 1.** Mean values ± SEM for FITC-sinistrin t_1/2_, serum creatinine, and urea for all groups at all time-points evaluated. An one-way ANOVA statistical test with Bonferroni *Post Hoc* analysis was applied to each data set to compare the values at baseline and at day 14. * P<0.05; ** P<0.01; *** P<0.001 one-way ANOVA with Bonferroni correction.

**Supplemental table 2.** Estimation of the extent of renal engraftment of CD133^+^ cells reported in a previous study^a^

^a^ See Ronconi et al., where 0.75 x 10^5^ PKH26-labelled CD133^+^ cells were injected into the tail vein of mice on the 1^st^ and 4^th^ day following the administration of adriamycin. On day 7, the total number of podocytes and proximal tubule cells (PTCs) were counted in histological sections by staining for podocin and LTL (Lotus tetragonolobus agglutinin). The proportion of podocin^+^ and LTA^+^ cells labelled with PKH26 was reported to be 11% and 7%, respectively. As indicated in the above table, the total number of podocytes and PTCs per mouse is estimated to be 3.6 x 10^5^ and 12.8 x 10^6^. Thus, 11% of podocytes and 7% of PTCs would be equivalent to ~0.4 x 10^5^ and ~0.9 x 10^6^ cells, giving a total cell number of 0.94 x 10^6^, which would amount to 63% of the injected cell dose (two administrations of 0.75x10^6^ cells). Even if none of the cells died, which would be unlikely, and all passed through the lungs into the left ventricle, an engraftment level of 63% does not seem feasible because the kidneys only receive 20% of the cardiac output.

1. Nicholas SB, Basgen JM, Sinha S. Using stereologic techniques for podocyte counting in the mouse: shifting the paradigm. *Am J Nephrol.* 2011;33 Suppl 1:1-7.

2. Zhai XY, Birn H, Jensen KB, Thomsen JS, Andreasen A, Christensen EI. Digital three-dimensional reconstruction and ultrastructure of the mouse proximal tubule. *Journal of the American Society of Nephrology : JASN.* 2003;14(3):611-619.

3. Sakamoto H, Sado Y, Naito I, et al. Cellular and subcellular immunolocalization of ClC-5 channel in mouse kidney: colocalization with H+-ATPase. *Am J Physiol.* 1999;277(6 Pt 2):F957-965.

4. Murawski IJ, Maina RW, Gupta IR. The relationship between nephron number, kidney size and body weight in two inbred mouse strains. *Organogenesis.* 2010;6(3):189-194.

5. Ronconi E, Sagrinati C, Angelotti ML, et al. Regeneration of glomerular podocytes by human renal progenitors. *Journal of the American Society of Nephrology : JASN.* 2009;20(2):322-332.

# Supplemental Videos

Supplemental video 1. Volume rendering of human CD133^+^ cell in lung tissue 1 hour after cell injection.

Volume rendering of rat lung sections show a human GFP^+^ CD133^+^ cell (green) labelled with PKH26 (red), surrounded by CD68^+^ cells (white). PKH26^+^ can be observed in the proximity of the human cell, co-localizing with CD68^+^ cells. Imaris software was used to generate volume rendering, starting from a stack of confocal fluorescence images (section thickness 6 µm).

## Supplemental video 2. Volume rendering of human CD133+ cell in lung tissue 24 hours after cell injection.

Volume rendering of rat lung sections show a fragment of a human GFP^+^CD133^+^ cell (green), surrounded by a cluster of CD68^+^ cells (white). PKH26^+^  (red) can be observed in the proximity of the human cell and within CD68^+^ cells. Imaris software was used to generate the volume rendering, starting from a stack of confocal fluorescence images (section thickness 6 µm).
